# Liver venous deprivation for resection of advanced hilar cholangiocarcinoma—a case report and review of the literature

**DOI:** 10.37349/etat.2022.00073

**Published:** 2022-02-28

**Authors:** Radoslava Stoyanova, Helmut Kopf, Wolfgang Schima, Wolfgang Karl Matzek, Alexander Klaus

**Affiliations:** 1 Department of Surgery, Barmherzige Schwestern Krankenhaus, 1060 Vienna, Austria; 2 Department of Diagnostic and Interventional Radiology, Barmherzige Schwestern Krankenhaus, 1060 Vienna, Austria; 3 Department of Diagnostic and Interventional Radiology, Göttlicher Heiland Krankenhaus, 1170 Vienna, Austria; Humanitas University, Humanitas Research Hospital, Italy

**Keywords:** Hilar cholangiocarcinoma, Klatskin, liver venous deprivation, portal vein embolization, future liver remnant, extended hepatectomy

## Abstract

Hilar cholangiocarcinoma is a rare primary malignancy associated with a dismal prognosis. Currently, complete extended right or left-sided hepatectomy is the primary curative therapy. Achieving a negative resection margin is associated with long-term survival and better quality of life, while post-hepatectomy liver failure (PHLF) due to insufficient liver remnant remains the most dreaded complication with a negative effect on overall survival. Precise preoperative management with sufficient future remnant liver (FRL) volume is the key to achieving good results in the treatment of bile duct carcinoma. To present a case report and a literature review for preoperative FRL optimization prior to major hepatectomies for hilar cholangiocarcinoma. Improvement of postoperative outcomes after extended liver resections in the case of hilar cholangiocarcinoma. A 62-year-old Caucasian woman with Lynch syndrome presented to our department with a hilar cholangiocarcinoma Bismuth type IIIa. The patient had an insufficient future liver volume for extended liver resection. She underwent preoperative preconditioning using a liver venous deprivation (LVD) and underwent two weeks later a right trisectorectomy without any interventional complications. Liver function remained stable postoperatively. The patient was discharged on the 20th postoperative day without major surgical post-operative complications or the need for readmission. LVD is a technically feasible, safe, and effective procedure to increase the FRL in a short period of time with low intra and post-operative complications and therefore improving the survival of patients with hilar cholangiocarcinoma.

## Introduction

Hilar cholangiocarcinoma accounts for less than 1% of all human malignancies [1], is commonly diagnosed in the advanced stage of the disease and has a dismal prognosis. Currently, complete extended right or left-sided hepatectomy is the primary curative therapy. Achieving a negative resection margin is associated with long-term survival and better quality of life [2]. Complications after hepatic resection occur in up to 40% of patients without cirrhosis, of which 20% are major complications with a mortality rate of 1–3% in high volume centers [3]. Post-hepatectomy liver failure (PHLF) remains the most dreaded complication in major hepatectomies reducing the overall prognosis and survival rates of bile duct carcinoma.

In 1990, Makuuchi et al. [4] proposed the use of portal vein embolization (PVE) to induce preoperative hypertrophy of the future remnant liver (FRL) in an attempt to increase the safety of major hepatectomy procedures for hilar bile duct carcinoma. PVE is considered preoperatively when FRL amounts to less than 25% of the standard liver volume or less than 40–45% in the case of steatotic or cirrhotic liver parenchyma. Either the left or right portal vein branches are embolized, leading to a redistribution of blood flow, causing hypertrophy in the contralateral hepatic segments to 30–40% of the total liver volume over 4–6 weeks [4]. Unfortunately, approximately 20% of patients undergoing PVE are not able to undergo hepatectomy due to inadequate hypertrophy [5].

Associating liver partition and portal vein ligation for staged hepatectomy (ALPPS) was first described in 2012 as an alternative procedure to exhibiting a greater increase in FRL in a shorter time, but it is associated with higher morbidity rates (33–58% for ALPPS *vs.* 16% after PVE) [5–6].

In 2016, Guiu et al. [7] assessed liver venous deprivation (LVD) with concurrent PVE and hepatic vein embolization (HVE) in 7 patients with an FRL increase of 64.3% by 3 weeks, with maximum liver function at 1 week and a 53.4% increase of FRL volume at 1 week. They reported 100% technical success, no patient mortalities, a resection rate performed in 6/7 patients and no incidence of PHLF [7].

According to this data, we decided to perform preoperative preconditioning using LVD in our patient.

We performed a retrospective analysis of the patient’s history and perioperative outcome. In addition, a literature review in PubMed using the keywords: Klatskin tumor, LVD, and trisectorectomy were conducted.

## Case report

**Figure 1.**
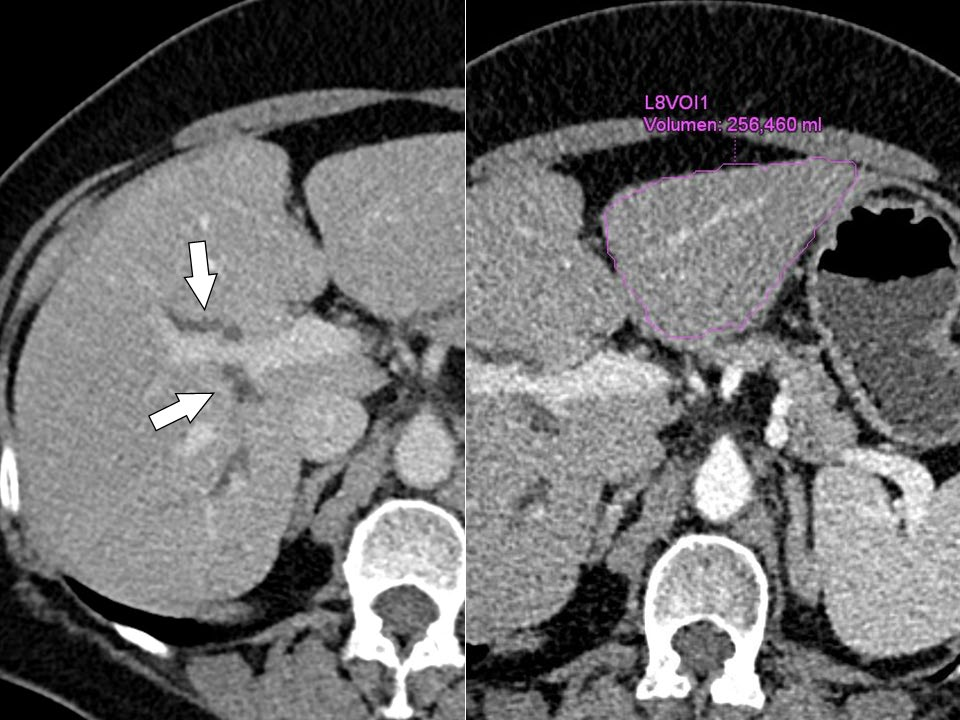
Contrast-enhanced MDCT (left) shows segmental biliary duct dilatation (arrows) in the right lobe, without demonstration of a tumor; (Right) of note is the small liver segments 2/3 with a volume of 256 mL. MDCT: multidetector CT

**Figure 2.**
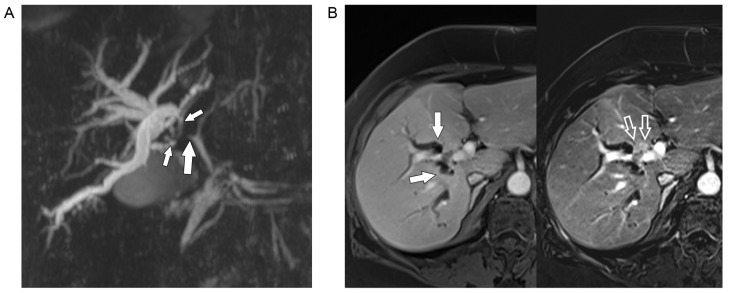
A. MRCP shows stenosis of the right hepatic duct (big solid arrow) extending into the segmental ducts of the right lobe (small solid arrows) with significant biliary dilatation; B. gadolinium-enhanced MRI (left) shows dilated segmental ducts (arrows) converging towards a faintly hyperintense mass anterior to the right portal vein. In the subtracted image (right) the small hypervascular tumor (open arrows) encroaching upon the right portal vein branch is much better seen

A 62-year-old Caucasian female patient was admitted to our surgical department with the diagnosis of hilar cholangiocarcinoma classified as Klatskin tumor Bismuth type IIIa. She had been diagnosed with Lynch syndrome [heterozygous mutation of DNA mismatch repair mutL homolog 1 (*MLH1*) gene], which leads to a higher risk of development of gastrointestinal, endometrial, urothelial, or pancreaticobiliary system cancers. Since 1997 several surgical procedures have been performed on this patient due to renal cell, urothelial, bladder carcinoma, and breast cancer. The patient was presumably tumor-free at the time of admission, but unfortunately, she had developed chronic renal insufficiency during the course of her therapies. She had undergone radiochemotherapy with curative intent for esophageal cancer staged cT4 N0 M0. Follow-up computed tomography (CT) of the chest, abdomen, and pelvis showed dilated intrahepatic bile ducts in the right lobe, but no definite tumor (Figure 1). Magnetic resonance cholangiopancreatography (MRCP) and contrast-enhanced magnetic resonance imaging (MRI) showed a hilar tumor, which compressed the right hepatic duct extending to the segmental ducts and showed contact with the right portal vein (Figure 2A, 2B). As no other signs of metastatic disease from esophageal cancer were present, a presumed diagnosis of cholangiocarcinoma classified as Klatskin tumor type 3A according to Bismuth-Corlette was made.

**Figure 3.**
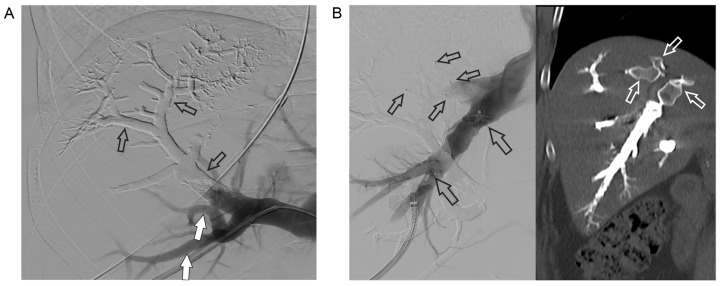
A. PVE: a part of the right portal vein and the branches of segments 7 and 8 (open arrows) are already filled with embolization material (Glubran 2/Lipiodol), in contrast, the percutaneous accessed branches of segments 5 and 6 are not embolized but opacified with contrast media at the time of DSA (open arrows); B. occlusion of the right hepatic vein (left): venogram of the percutaneously punctured right hepatic vein. An Amplatzer vascular plug 2 with 16 mm diameter (large open arrows) has already been detached. Small open arrows point at 2 other vascular plugs detached in two (non-opacified) tributaries of the right hepatic vein. Post-intervention CT in the coronal plane (right) shows the vascular plugs (open arrows) and the right hepatic vein completely filled with embolization material. DSA: digital subtraction angiography

We decided to perform a preoperative LVD because of the insufficient FRL (256 mL for the patient’s weight of 74 kg). The right portal branches for segments 5–8 were percutaneously punctured and embolized with a mixture of Glubran 2 (GEM, Italy) and Lipiodol (Guerbet, France) at a ratio of 1:5 (Figure 3A). In addition, the right liver vein was also percutaneous accessed and all three branches of the right liver vein were occluded using three Amplatzer vascular plugs (Abott Cardiovascular, USA) and embolized with Glubran 2/Lipiodol. Plugs were positioned to leave access to 1–2 cm of the distal end of the hepatic vein for easy dissection and clamping during the operation. The LVD procedure was finished uneventfully (Figure 3B).

After the intervention, the gamma-glutamyl transferase (GGT) was lightly elevated (66–80 U/l, normal range 0–39 U/l) without any other changes in liver function tests. The patient was discharged on the 3rd day after the intervention. Two weeks later she was readmitted for follow-up CT and measurement of the FRL volume.

**Figure 4.**
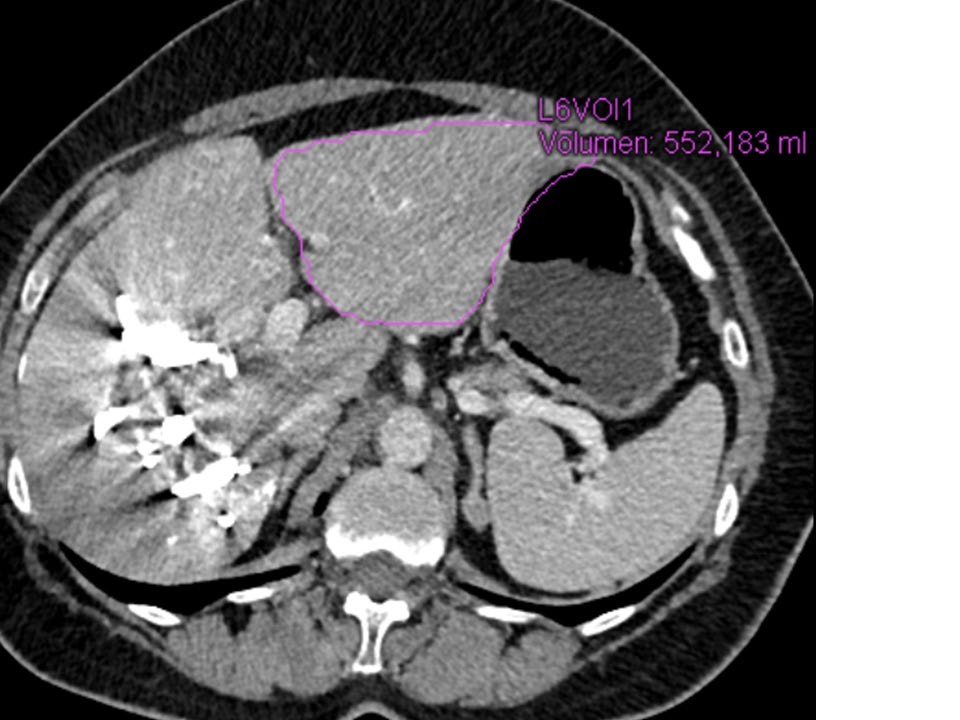
MDCT scan two weeks after LVD procedure demonstrates significant hypertrophy of segments 2/3 (FRL of 552 mL)

**Figure 5.**
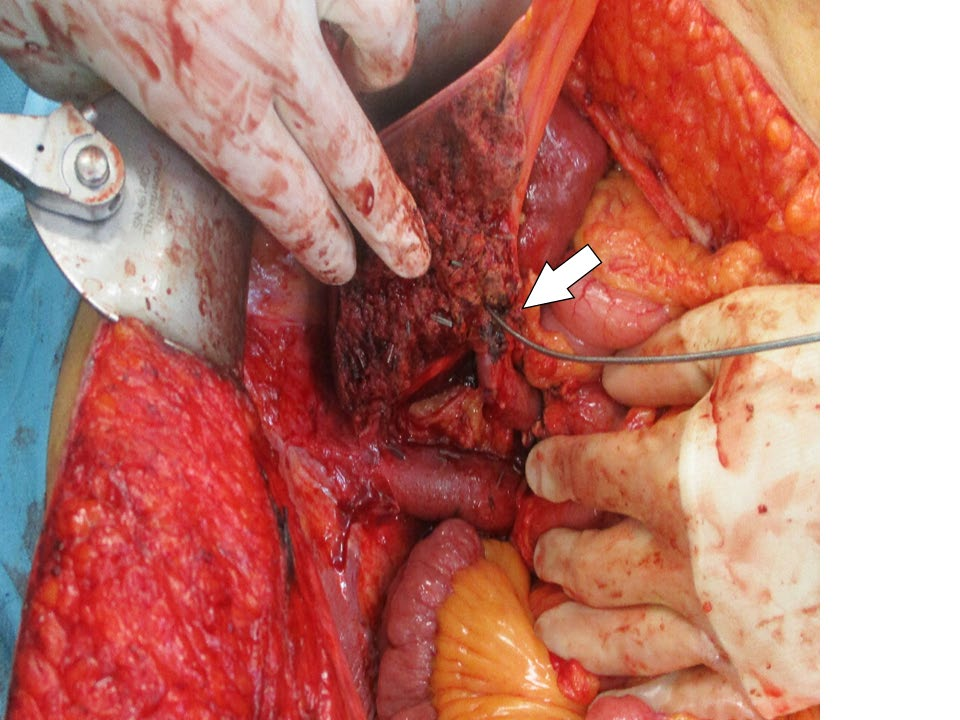
Intraoperative view of the transected liver parenchyma with the left hepatic duct cannulated (arrow)

The contrast-enhanced CT scan showed shrinkage of the embolized right liver as well as significant hypertrophy of segments 2 and 3 with sufficient remnant liver parenchyma (552 mL) (Figure 4). To proceed with an extended right hepatectomy (Figure 5). A functional maximum liver function capacity (LiMax) test (i.e., dynamic liver function test based on the metabolism of ^13^C-methacetin by the liver-specific cytochrome P450 1A2 system) after LVD was normal and showed even better results in the second week after the intervention compared to the first one.

A right trisegmentectomy with Roux-Y hepatico-jejunostomy was performed (Figure 5). The operative time was 254 min with a blood loss of 700 mL. The anastomosis was protected with a Neuhaus closed loop drain. A frozen section of the bile duct resection margin was negative for tumor cells. The patient was transferred to the intensive care unit (ICU) department for 3 days and recovered with no signs of PHLF. On the 7th postoperative day (POD) an intravenous antibiotic therapy was administered due to a fever and elevation of the blood infection parameters (Clavien-Dindo grade 2). The patient’s recovery was uneventful and a cholangiography using the Neuhaus drain was performed on day 17. There were no signs of anastomotic leakage and the drain was removed.

The patient was discharged on day 20. The final histological result confirmed a medium-highly differentiated cholangiocarcinoma stage pT2b L0 V0 pN0 (0/1) G2 with clear surgical margins (R0). MDCT follow-up every 3 months for the next two years has been scheduled.

## Discussion

Very little scientific literature has been published about optimizing FRL using LVD before extended liver resections in cases of hilar cholangiocarcinoma. Our research in PubMed using the keywords Klatskin tumor and LVD showed only 4 results (retrospective studies and small series with less than 51 patients), 3 of them published by one author [7–10]. In one study, Hocquelet et al. [8] compared an estimated FRL growth following PVE or LVD before surgery for Klatskin tumors in patients with preoperative biliary drainage. This study investigated 12 participants divided into two groups (6 patients underwent LVD and 6 underwent PVE alone before hepatectomy). Biliary drainage was placed in all patients preoperatively either by endoscopy or by a percutaneous approach. The authors reported a significantly higher median FRL ratio in the LVD group and a trend towards earlier discharge after surgery. In the two other studies, Guiu et al. [7, 9] described not only a fast but also safe FRL hypertrophy after LVD [7] and a very rapid increase in liver function after extended LVD [eLVD, i.e., combination of right PVE and right (accessory right) and middle HVE] [9] using ^99m^Tc-mebrofenin hepatobiliary scintigraphy and CT for functional and morphologic assessment, respectively.

In our patient, FRL volume increased from 256 mL to 552 mL within two weeks after LVD. Liver capacity was measured using a LiMax test. The test was normal after LVD and showed increasing liver capacity (in the first-week value of 349 μg/h per kg and in the second week a value of 444 μg/h per kg with a normal range of > 315 μg/h per kg). We found no other studies in PubMed describing the usage of a LiMax test for the evaluation of liver function after LVD. This test does not involve ionizing radiation and could be repeated many times, bearing some advantages for the patient in comparison with ^99m^Tc-mebrofenin hepatobiliary scintigraphy.

In another study, Guiu et al. [10] confirmed the safety, greater FRL volume, and function increase after LVD compared with PVE. Unfortunately in this study patients with Klatskin tumors were excluded.

The DRAGON-1 international control trial [11] published in 2021 is the biggest collaborative group analysis nowadays, which assessed hypertrophy of the FRL after PVE or LVD and reviewed improvement of resectability in 199 patients (30 patients with hilar cholangiocarcinoma included) treated in 7 centers. It shows statistically significantly better hypertrophy and an improved resectability in the LVD group than in the PVE group. Major complications and 90-day mortality were comparable between the groups [11].

In spite of the limitations of these studies, all of them showed similarly low mortality/morbidity rates using the LVD technique compared to PVE, while LVD demonstrated a very fast increase in FRL volume and liver function over a shorter period of time. These results could be compared to the ALPPS technique. However, LVD therapeutic strategy avoids a two-stage surgery and the high morbidity and mortality rates reported after ALPPS [12, 13]. The good early postoperative outcome of our patient is in line with the results on LVD reported in the literature.

In conclusion, LVD is a feasible and well-tolerated procedure, which provides fast and significant hypertrophy of the FRL before major hepatectomy in the case of a Klatskin tumor. This technique needs to be further evaluated in larger studies.

## Abbreviations

ALPPS: associating liver partition and portal vein ligation for staged hepatectomy
CT: computed tomography
FRL: future remnant liver
LiMax: maximum liver function capacity
LVD: liver venous deprivation
PHLF: post-hepatectomy liver failure
PVE: portal vein embolization

## Declarations

### Copyright

© The Author(s) 2022.
